# Value of plasma homocysteine to predict stroke, cardiovascular diseases, and new-onset hypertension: A retrospective cohort study

**DOI:** 10.1097/MD.0000000000021541

**Published:** 2020-08-21

**Authors:** Yuanyuan Feng, Kai Kang, Qiqi Xue, Yafen Chen, Wei Wang, Jiumei Cao

**Affiliations:** Department of Geriatrics, Ruijin Hospital, Shanghai Jiaotong University School of Medicine, Shanghai, China.

**Keywords:** cardiovascular disease, homocysteine, hypertension, predictive value, stroke

## Abstract

The influences of hyperhomocysteinemia on cardiovascular diseases (CVDs), stroke and new-onset hypertension are unclear. The aim of the study is to explore the associations of homocysteine levels with stroke, CVDs, and new-onset hypertension in Chinese individuals.

This retrospective cohort study included outpatients and inpatients from the Department of Geriatrics at Ruijin Hospital affiliated to Shanghai Jiaotong University School of Medicine from January to December 2000. They were divided based on their homocysteine (Hcy) levels in 2000: Q1 (<10 μmol/L), Q2 (10–15 μmol/L), and Q3 (>15 μmol/L) and according to whether they had hypertension at baseline. Information about stroke, mortality and major adverse cardiac events, and newly onset hypertension was gathered in December each year until 2017. The effects of Hcy levels on the risk for stroke and CVDs among all patients, and new-onset hypertension among patients without hypertension at baseline were evaluated.

After adjustment for confounders, compared with the Q1 group (Hcy <10 μmol/L), when the Hcy increased to 10 to 15 μmol/L, the risks for stroke, CVDs, and new-onset hypertension significantly increased, and the hazard ratio and 95% confidence interval were 2.02 (1.35–3.05, *P* = .001), 2.22 (1.32–3.76, *P* = .003), and 7.20 (4.52–11.48, *P* < .001), respectively. Hcy improved the predictive capability of traditional risk factors for stroke. The optimal cut-off value of Hcy for predicting stroke was 13.4 μmol/L (sensitivity: 70.9%, specificity: 62.2%).

Hcy 10 to 15 μmol/L is significantly associated with the risks for stroke, mortality and major adverse cardiac events, and hypertension. The best cut-off point of Hcy for predicting stroke is 13.4 μmol/L.

## 1 Introduction

Cardiovascular diseases (CVDs) are among the most important threats to public health worldwide. There were 2.4 million deaths due to atherosclerotic CVDs in 2016 in China, representing 61% of deaths due to CVDs and 25% of all deaths.^[1]^ Furthermore, the incidence and mortality of CVDs has been increasing from 1990 to 2016 in China,^[1]^ probably due to lifestyle changes associated with the new economy.^[2]^ Hypertension is an important independent risk factor for CVDs.^[3]^ In China, 23.3% of Chinese adults (about 244 million persons) have hypertension and the condition is adequately controlled in only 15% of them.^[4]^

In addition to improving lifestyle and adopting drug therapies to prevent these diseases, the relationship between homocysteine (Hcy) and these diseases is gradually attracting attention.^[5–7]^ Hcy is a sulphur-containing amino acid produced by methionine metabolism and is mainly cleared through the kidneys. The reference values of Hcy will be different in different races and populations, and will vary among different measurement methods. Typically, the normal reference range of Hcy is 5 to 15 μmol/L. Therefore, the definition of hyperhomocysteinemia (Hhcy) is controversial, but it is generally defined as plasma Hcy ≥15 μmol/L,^[8,9]^ but it also has been known to be defined as plasma Hcy ≥10 μmol/L.^[10–12]^ There is a large amount of epidemiological data demonstrating that elevated plasma Hcy is an independent risk factor for CVDs,^[13–15]^ even when the plasma Hcy levels are only slightly increased (10–15 μmol/L).^[16]^ Nevertheless, there are only a few long-term follow-up cohort studies available and there are currently no cohort studies that assessed the influence of Hhcy on cardiovascular events in patients with or without hypertension.

In recent years, many studies on different populations assessed the relationship between Hcy and hypertension and showed that Hcy is associated with the occurrence of hypertension and may interact with hypertension, affecting together the incidence of CVDs.^[5,17]^ In China, due to the lack of folic acid and B vitamin intake in the diet and a high mutation rate of the methylenetetrahydrofolate reductase gene (the C677T single nucleotide polymorphism), which can cause an increase in Hcy, the proportion of patients with hypertension and Hhcy, which is known as H-type hypertension, is higher than that in other populations.^[18–20]^ A study showed that Hhcy in Chinese hypertensive patients is associated with the risk of ischemic stroke, but not with the risk of coronary heart disease.^[21]^ In Chinese populations, multivitamin supplementation containing folic acid decreases the risk of stroke, but not of coronary heart disease.^[22–24]^

Therefore, the aim of the present study was to explore the associations of Hcy with stroke and CVD in all patients, and with new-onset hypertension in patients without hypertension at baseline. The results could provide important insights in the pathogenesis of CVDs and hypertension associated with Hhcy, and may help in the management of the patients.

## 2 Material and methods

### 2.1 Study population

This retrospective cohort study included outpatients and inpatients from the Department of Geriatrics at Ruijin Hospital affiliated to Shanghai Jiaotong University School of Medicine from January to December 2000. The study was approved by the ethics committee of Ruijin Hospital Affiliated to Shanghai Jiaotong University School of Medicine. Written informed consent was waived by the committee.

The inclusion criteria were:

1. no new-onset stroke and CVDs in the past 1 year,
2. no history of secondary hypertension,
3. glomerular filtration rate >30 ml/min/1.73 m^2^, and
4. did not take folic acid, vitamin B12, or other drugs that can influence the Hcy levels for the past 1 year.

The exclusion criteria were:

1. heart failure,
2. systolic blood pressure (SBP) >185 mm Hg or diastolic blood pressure (DBP) >110 mm Hg,
3. severe liver or kidney dysfunction needing replacement therapy,
4. recent acute infection,
5. family history of mental illness or use of psychotropic, or
6. lost to follow-up.

### 2.2 Data collection

Clinical data (including demographic characteristics and chronic disease history) and results of laboratory test (including blood glucose, blood lipids, liver and kidney function, and Hcy) were collected from an electronic clinical information system at Ruijin Hospital at baseline. The patient data included age, gender, body mass index (BMI), history of smoking, history of drinking, history of diabetes, stroke history, blood pressure, family history of hypertension, and family history of stroke. Kidney dysfunction was defined as glomerular filtration rate <90 ml/min·1.73 m^2^.^[25]^

All patients underwent biochemistry examination, including fasting plasma glucose, triglycerides, total cholesterol, high-density lipoprotein cholesterol (HDL-C), low-density lipoprotein cholesterol (LDL-C), alanine aminotransferase, and aspartate aminotransferase. Plasma folic acid and vitamin B12 levels were measured by electro-chemiluminescence method with an Elecsys 2010 system (Roche Diagnostics, Basel, Switzerland).

Plasma Hcy levels were determined at baseline by an enzyme-linked immunosorbent assay (ELISA) using a coda automatic plate type enzyme-labeled instrument (Bio-Rad, Hercules, CA). The patients were divided into 3 groups according to the Hcy level, the Q1 group (Hcy < 10 μmol/L), Q2 group (Hcy = 10–15 μmol/L) and Q3 group (Hcy > 15 μmol/L).^[8–12]^

### 2.3 Outcomes and follow-up

The primary outcome was the relationship between different Hcy levels and the risk for stroke, CVDs, and new-onset hypertension. The secondary outcome was the best cut-off value of Hcy for predicting stroke, CVDs, and newly onset hypertension.

All subjects participated in physical examinations at the Ruijin Hospital every year and they would see a doctor in our hospital if there was any discomfort. In addition, almost every surviving patient accepted follow-up for blood pressure and other examinations every 3 months for new-onset stroke, CVDs, and new-onset hypertension from the electronic clinical information system at Ruijin Hospital in December of each year until 2017.

New stroke and CVDs were recorded in all patients. Stroke was defined as a focal neurological deficit lasting >24 hours caused by intracerebral hemorrhage or infarction, and with computed tomography or magnetic resonance imaging evidence.^[26]^ The CVDs included angina, myocardial infarction, percutaneous coronary intervention, and coronary artery bypass graft.^[7,13,27]^ For patients without hypertension at baseline (n = 597), the incidence of new-onset hypertension was collected. Hypertension was defined as SBP ≥ 140 mm Hg or DBP ≥90 mm Hg.^[28]^ Blood pressure measurements were performed in accordance with the National Guidelines for the Prevention and Control of Hypertension of China Basic Public Health Services.^[28]^

### 2.4 Statistical analysis

SPSS 20.0 (IBM, Armonk, NY) was used to manage and analyze the data. The continuous variables are described as mean ± standard deviation or median (interquartile range) after having been tested for normal distribution using the Kolmogorov-Smirnov test and were analyzed using the Student *t* test or ANOVA with the SNK post hoc test, as appropriate. Categorical variables are expressed as percentages, and were analyzed using the chi-square test. The occurrence of stroke, CVDs and new-onset hypertension were estimated using the Kaplan-Meier method and compared using the log-rank test. The receiver operating characteristic (ROC) curve was plotted and determined the optimal cut-off values of Hcy with the highest Youden index for predicting stroke, CVDs, and new-onset hypertension. The Cox proportional hazards regression model was applied to calculate the hazard ratio and corresponding 95% confidence interval (CI) to explore the effects of Hcy levels on the risk for stroke, CVDs, and new-onset hypertension. The dependent variable was the occurrence of stroke, CVDs, or new-onset hypertension. The variables that were significant (*P* < .05) in univariable analyses were entered in the Cox model (enter method).

## 3 Results

### 3.1 Baseline demographic and clinical characteristics

A total of 385 patients did not meet the inclusion criteria and 102 (7.7%) patients lost to follow-up were excluded from the analysis. Therefore, a total of 1226 patients were included in the final study cohort. The age range of the included patients was 42 to 97 years, with an average age of 70.2 ± 9.8 years. The study population included 1058 men (86.3%) and 168 women (13.7%). The 1226 subjects were directly divided into 3 groups according to the Hcy levels measured at baseline. On the other hand, the 1226 subjects were also stratified according to hypertension at baseline.

Table 1 presents the characteristics of the participants. There were 414 subjects (33.8%) in the Q1 group (Hcy <10 μmol/L), 393 (32.1%) in the Q2 group (Hcy = 10–15 μmol/L), and 419 (34.2%) in the Q3 group (Hcy >15 μmol/L). Mean age was 68.2 ± 11.1 years in the Q1 group, 71.2 ± 9.0 years in the Q2 group, and 71.4 ± 8.7 years in the Q3 group (*P* < .01). The percentages of males were 343 (82.9%), 352 (89.6%), and 363 (86.6%) in the Q1, Q2, and Q3 groups (*P* = .02), respectively. Meanwhile, compared with those in the Q1 group, those with a higher Hcy level had significantly higher rates of smoking, hypertension, diabetes mellitus, and renal function decline, while the levels of folic acid and vitamin B12 decreased gradually (all *P* < .05).

**Table 1:**
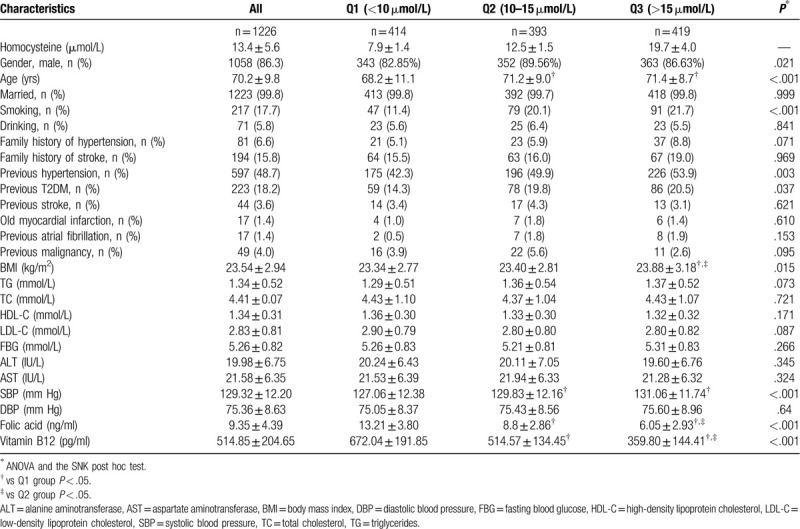
Characteristics of the participants.

As presented in Table 2, when stratified according to hypertension, the smoking rate, folic acid levels, and vitamin B12 level distributions among the hypertension subgroup of the Q1, Q2, and Q3 groups were significantly different (all *P* < 0.05). In the nonhypertension subgroup, significant differences in age, smoking rate, folic acid, and vitamin B12 were observed among the Hcy groups.

**Table 2:**
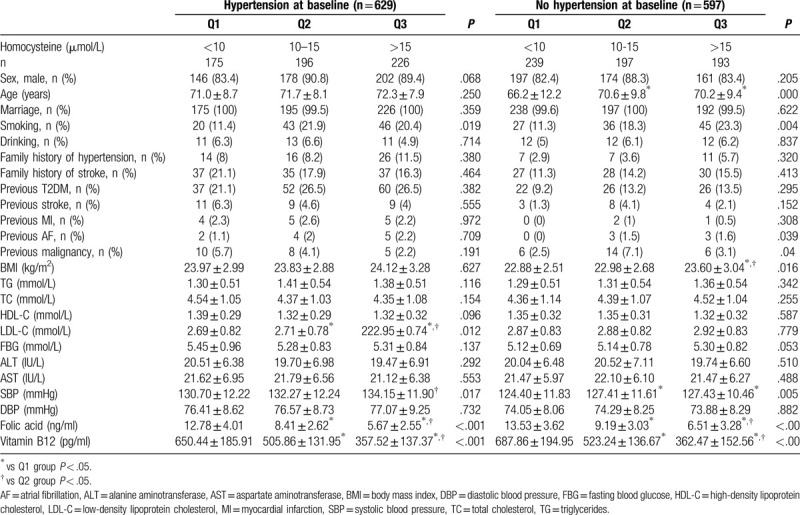
Baseline characteristics according to hypertension.

#### 3.1.1 Stroke, CVDs, and newly onset hypertension

**Figure 1:**
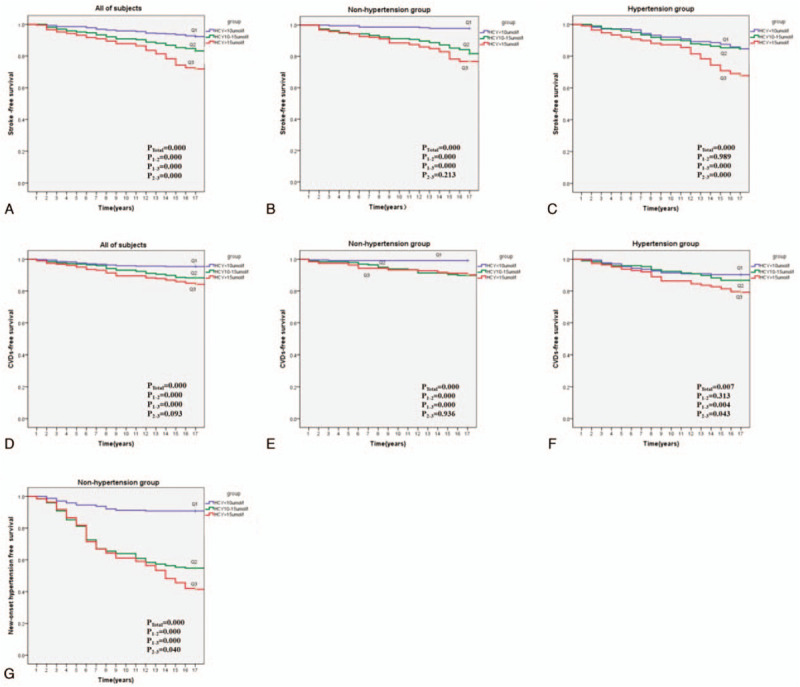
Kaplan Meier analysis of stroke, CVDs, and new-onset hypertension according to different homocysteine groups. (A–C) Kaplan Meier analysis of stroke according to different homocysteine groups in all of subjects (A), in the nonhypertension subgroup (B), and in the hypertension subgroup (C). Log rank test *P* < .001. (D-F) Kaplan Meier analysis of CVDs according to different homocysteine groups in all of subjects (D), in the nonhypertension subgroup (E), and in the hypertension subgroup (F). (G) Kaplan Meier analysis for new-onset hypertension according to different homocysteine groups in the non-hypertension subgroup. CVDs = cardiovascular diseases.

After 17 years of follow-up in the 1226 participants, we recorded a total of 237 strokes, which included 141 in the hypertension subgroup and 96 in the nonhypertension subgroup. As shown in Figure 1A to C, the cumulative incidence of stroke increased significantly with the Hcy levels (*P* < .001), as well as in the nonhypertension and hypertension subgroups.

Among all subjects, 145 (99 hypertensive subjects and 46 nonhypertensive subjects) had CVD during the 17 years of follow-up. For both subgroups, the Q2 subjects had a higher risk for CVD compared with Q1; Q3 had a significantly higher risk (Fig. 1D–F).

There were 236 cases of new-onset hypertension documented among the 629 nonhypertensive subjects at baseline (Fig. 1G). The risk of new-onset hypertension increased with Hcy levels (all *P* < .01).

#### 3.1.2 Receiver operating characteristic analysis of the optimal cut-off value for predicting outcomes according to Hcy

**Figure 2:**
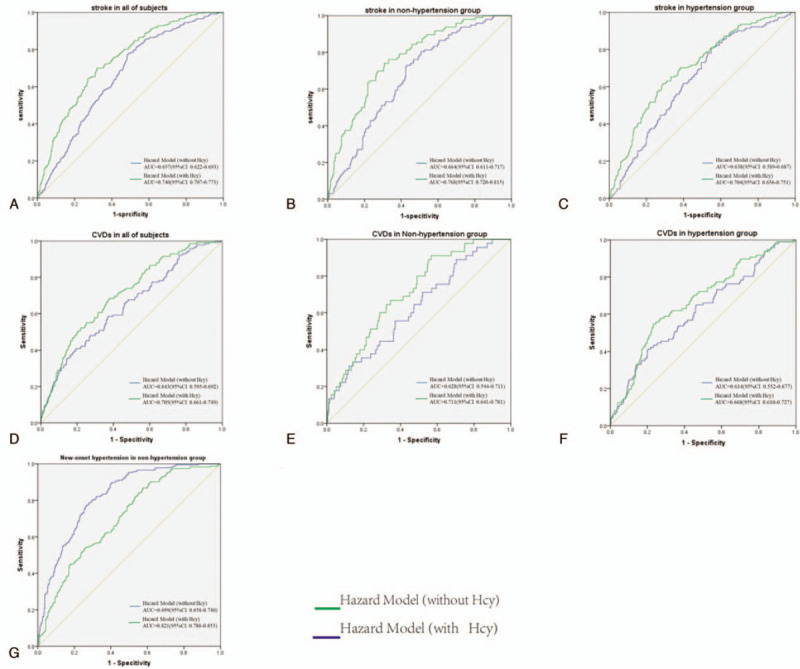
ROC analysis for predicting stroke, CVDs, and new-onset hypertension. (A–C) ROC analysis for predicting stroke in all subjects (A), in the nonhypertension subgroup (B), and in the hypertension subgroup (C) during the 17-year follow-up. (D–F) ROC analysis for predicting CVDs in all subjects (D), in the nonhypertension subgroup (E), and in the hypertension subgroup (F) during the 17-year follow-up. (G) ROC analysis for predicting new-onset hypertension in the non-hypertension subgroup. CVDs = cardiovascular diseases, ROC = receiver operating characteristic.

Because the occurrence of stroke is influenced by multiple factors, we established a risk factor model for stroke with or without Hcy. As shown in Figure 2A to C, Hcy could improve the predictability of the risk factor model for stroke in all participants and in the nonhypertension and hypertension subgroups (Fig. 2A–C), with areas under the ROC curves (AUCs) of 0.740 (95% CI 0.707–0.773) vs 0.657 (95% CI 0.622–0.740), 0.768 (95% CI 0.720–0.815) vs 0.664 (95% CI 0.611–0.717), and 0.704 (95% CI 0.656–0.751) vs 0.638 (95% CI 0.589–0.687), respectively. In addition, to a certain extent, Hcy could increase the predictability of the risk factor model for CVDs and hypertension (Fig. 2D–G). In addition, the optimal cut-off values were determined using the Youden index. The corresponding sensitivities, specificities, Youden indexes, and hazard ratios (HRs) are presented in Table 3.

**Table 3:**
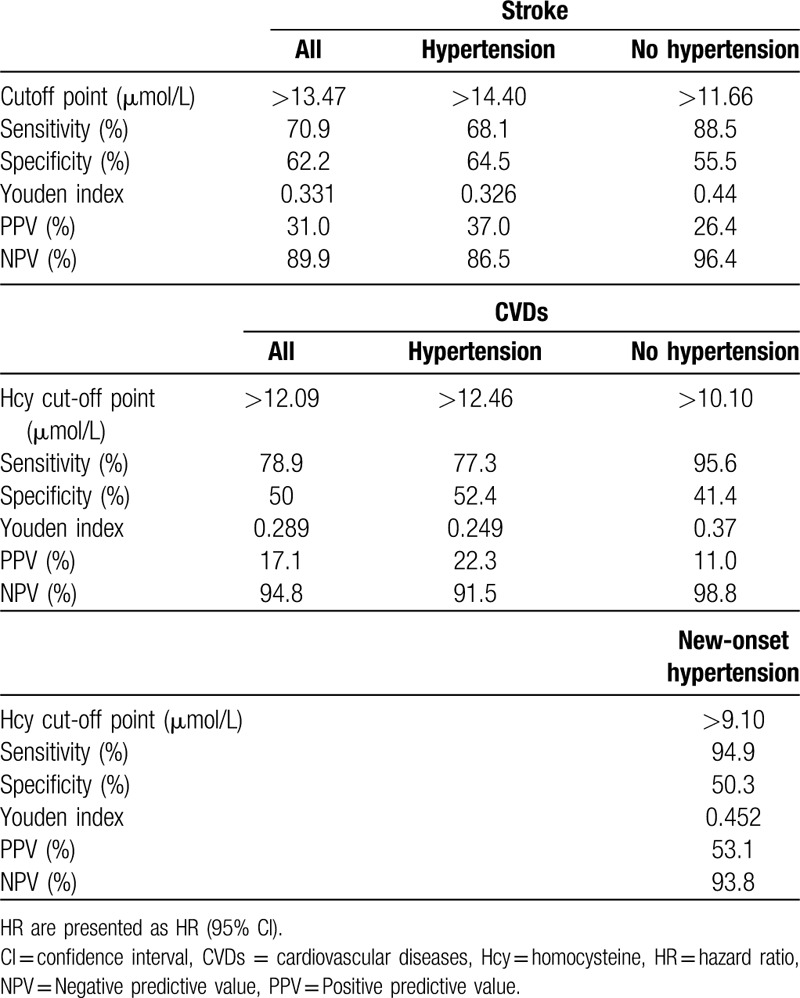
Results for measurement cut-off value of Hcy in the prediction for stroke, CVDs, and new-onset hypertension based on the ROC analysis.

#### 3.1.3 Univariable and multivariable Cox regressions for stroke, CVDs, and new-onset hypertension

Unadjusted and adjusted HRs for stroke and CVDs of the whole cohort are shown in Table 4. Variables that were significant (*P* < .05) in univariable analyses were included in the multivariable analysis. The multivariable analysis showed that age (HR = 1.04, 95% CI: 1.02–1.06, *P* < .001), Hcy (HR = 1.09, 95% CI: 1.07–1.11, *P* < .001), and history of hypertension (HR = 1.38, 95% CI: 1.04–1.81, *P* = .023), were independently associated with stroke. The second multivariable analysis showed that Hcy (HR = 1.07, 95% CI: 1.04–1.10, *P* < .001), history of diabetes (HR = 1.78, 95% CI: 1.22–2.59, *P* = .003), history of hypertension (HR = 1.70, 95% CI: 1.16–2.47, *P* = .006), and BMI (HR = 1.08, 95% CI: 1.02–1.13, *P* = .006) were independently associated with CVDs.

**Table 4:**
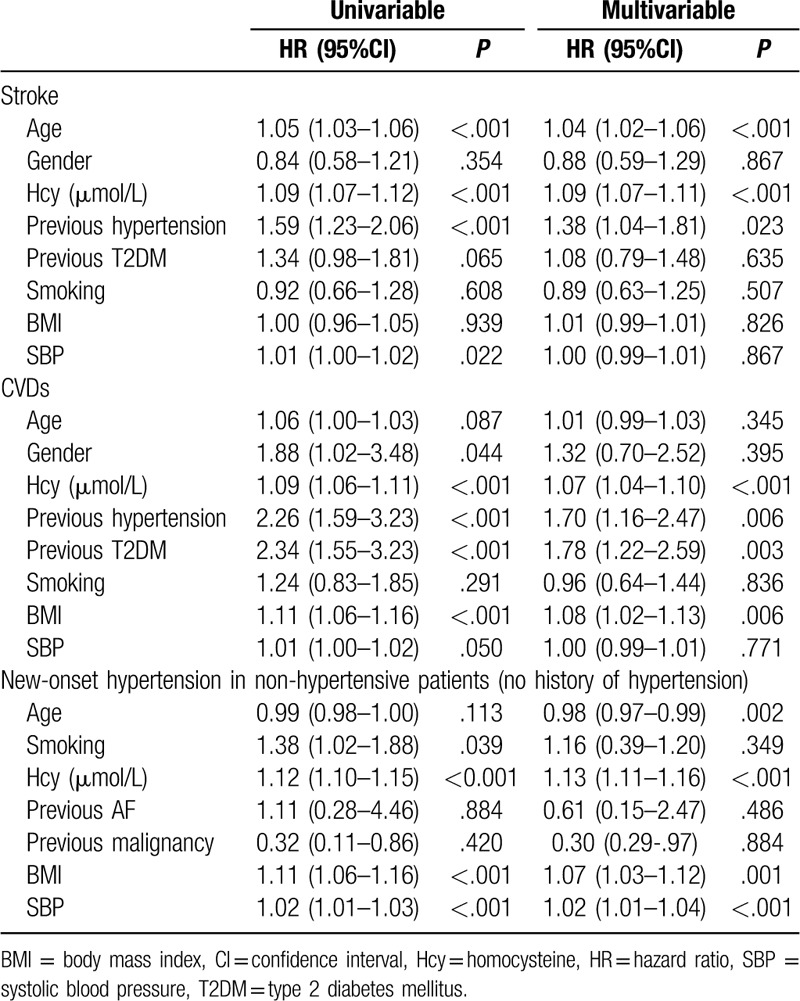
Univariable and multivariable Cox proportional hazard regression analyses of stroke and CVDs according to Hcy in all subjects.

For all patients, the analyses were adjusted for age, smoking, previous AF, previous malignancy, BMI, and SBP. In the hypertension subgroup, the analyses were adjusted for smoking, LDL, and SBP. In the non-HP subgroup, the analyses were adjusted for age, smoking, previous malignancy, previous AF, BMI, and SBP.

Table 5 shows the HRs from the subgroup analysis of the hypersensitive population after adjustments for smoking, LDL, and SBP. The risk for stroke and CVDs was increased (*P* < .05) in the Q3 group (adjusted HR [95% CI]: 2.12 [1.39–3.24] and 2.24 [1.29–3.88], respectively), whereas no statistically significant increase was found in the Q2 group (Table 5). On the other hand, in the non-hypertension subgroup, after adjustments for age, smoking, previous malignancy, previous AF, BMI, and SBP, and using Hcy < 10 μmol/L as the reference group, the risk for stroke, CVD, and new-onset hypertension was significantly increased in Q2 and Q3 subjects (*P* < .05).

**Table 5:**
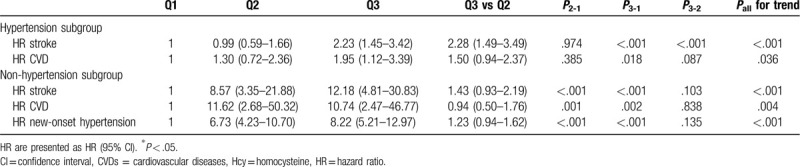
Cox proportional hazard regression analyses of stroke and CVDs for different levels of Hcy in subgroups.

## 4 Discussion

No cohort studies assessed the influence of Hhcy on cardiovascular events, stroke, and new-onset hypertension in Chinese subjects. Therefore, this study aimed to explore the associations of Hcy levels with stroke and CVDs in Chinese patients, and new-onset hypertension in patients without hypertension at baseline. The study, which included 1226 subjects with a 17-year follow-up, provides evidence that there is a significant association between elevated plasma Hcy (>10 μmol/L) and the risk of stroke, CVD, and new-onset hypertension among middle-age and old patients. Of note, we found that Hcy and hypertension jointly affected the risk of stroke and CVD. In addition, we attempted to provide a more accurate definition of Hhcy using ROC curves in order to improve its predictive capability for the risk of CVD.

There are few cohort studies with a long-term follow-up in Asian populations,^[29–32]^ and their conclusions are controversial. An increase in plasma Hcy levels is associated with a variety of vascular diseases.^[5,6,31]^ The possible mechanisms of vascular injury involve oxidative stress interfering with the balance of the nitric oxide synthase system, immune and inflammatory responses, dyslipidemia, and coagulation dysfunction.^[33–37]^ A combination of multiple mechanisms eventually leads to the development of atherosclerosis and CVD.

Stroke is the second leading cause of death in the world, and it is also one of the most common chronic noncommunicable diseases in China.^[38,39]^ In addition to the traditional risk factors of stroke (such as blood lipids, blood pressure, and smoking), a number of studies have proposed that elevated plasma Hcy is closely related to stroke risk.^[21,29,40,41]^ Similarly, the findings from some cross-sectional studies indicated that plasma Hcy levels in stroke patients are significantly higher than in individuals without stroke.^[42,43]^ Recently, a number of studies have suggested that reducing the level of Hcy can effectively decrease the risk of stroke, which indirectly indicates that Hcy is a risk factor for stroke. In 2015, the China Stroke Primary Prevention Trial (a randomized, double-blind clinical trial) reported that among hypertensive adults without a history of stroke or myocardial infarction, enalapril-folic acid significantly reduced the risk for first stroke compared with enalapril after a median treatment duration of 4.5 years.^[44]^ Nevertheless, there are still some studies indicating that Hcy-lowering therapy has no significant effect on reducing the risk of stroke.^[23]^ To evaluate the association between Hcy and stroke risk, we conducted an observational study with a 17-year follow-up. As hypothesized, among the 1226 subjects, after adjustment for confounding factors, the risk for stroke (HR = 2.02) in subjects with Hcy of 10 to 15 μmol/L was higher than in subjects with Hcy < 10 μmol/L, and subjects with Hcy > 15 μmol/L had an HR of 3.44. Moreover, Hcy of 10 to 15 μmol/L was found to be independently associated with the risk of stroke in the nonhypertensive subjects. In the hypertension subgroup, the risk for stroke was significantly increased in the Hcy > 15 μmol/L group. Finally, ROC curve analysis was performed to evaluate the value of Hcy for predicting stroke. The results indicated that Hcy can significantly improve the predictive capability of traditional risk factors for stroke. The Youden index indicated that Hcy > 13.5 μmol/L could be considered a promising marker for the prediction of stroke, with 70.9% sensitivity and 62.2% specificity. Nevertheless, the mechanisms of Hcy in promoting stroke are still unclear. The most likely causes are cerebral vascular injury and atherosclerosis, but further research is needed.

CVDs impose an enormous financial burden to the society and is one of the main causes of death worldwide.^[1]^ In addition to the traditional risk factors such as hyperlipidemia, smoking, hypertension, and diabetes, 15% to 20% of patients with CVD do not have these traditional risk factors and are not identified in time to prevent a primary event, thereby missing the opportunity for primary prevention. For these patients, Hcy can be considered to be one of the important potential risk factors of CVD.^[45]^ Ma et al^[46]^ showed that high levels of Hcy are significantly correlated with CAD categories (*r* = 0.286, *P* < .001). Compared to patients with stable angina pectoris, patients with acute myocardial infarction and unstable angina pectoris had higher Hcy levels by approximately 4 to 5 μmol/L.^[46]^ In line with these results, Fu et al^[47]^ reported that Hhcy plays a significant role in the prediction of long-term clinical outcomes, including all-cause mortality and major adverse cardiac events, in Chinese octogenarians with acute coronary syndrome. In the present study, by observing the occurrence of cardiovascular events at different Hcy levels over 17 years, we concluded that mildly elevated Hcy (>10 μmol/L) increased the risk for CVD events. For the nonhypertensive subjects, the risk for CVD was greater in those with Hcy of 10 to 15 μmol/L, for whom the predictive cut-off value was 10.10 μmol/L, but Hcy > 15 μmol/L was still a risk factor for CVD events in hypertensive patients. Taken together, the results suggest that Hcy > 10 μmol/L may be helpful for assessing the risk for CVDs and the need for Hcy-lowering therapy.

Furthermore, we also studied the relationship between Hcy levels and the risk for new-onset hypertension in the nonhypertension subgroup. The result showed that Hcy > 10 μmol/L significantly increased the risk for new-onset hypertension. ROC curve analysis indicated that the predictive cut-off value of Hcy for new-onset hypertension was 9.10 μmol/L with 94.9% sensitivity and 50.3% specificity. The pathogenesis of essential hypertension involves both genetic and environmental factors, and aside from well-established traditional predisposing factors such as obesity, alcohol consumption, high sodium and/or low potassium diet, lack of physical exercise and other environmental factors for the occurrence and development of hypertension, the role of Hcy has attracted an increasing amount of attention.^[48]^ Elevated Hcy can increase oxidative stress, decrease vasodilation by nitric oxide, and stimulate the proliferation of vascular smooth muscle cells. As a result, blood pressure increases.^[18,20]^ Many studies have shown that Hcy is associated with elevated blood pressure.^[5,17,18,20]^ For every 5 μmol/L increase in Hcy, the SBP and DBP increase by 0.7/1.2 mm Hg and 0.5/0.7 mm Hg in women and men, respectively.^[49]^ In addition, in 2016, a meta-analysis of 11 published epidemiological studies demonstrated that Hcy contributed to the risk of essential hypertension (OR = 1.36, 95% CI: 1.02–1.80, random-effects model). In summary, the early detection of elevated Hcy and timely intervention against elevated Hcy may provide a new therapeutic strategy to help strengthen primary and secondary prevention of essential hypertension and its complications.

This study shows that Hcy can serve as a marker for predicting risks for stroke, CVDs, and hypertension. MicroRNAs – a class of small, endogenous, single-stranded, non-coding RNAs – have also been found to be innovative biomarkers for facilitating diagnosis and predicting prognosis of stroke, CVD, and hypertension.^[50–52]^ In comparison, microRNAs might offer several advantages over Hcy. Firstly, microRNAs are comparatively stable in both plasma and serum,^[53]^ whereas Hcy can be easily affected by a range of factors. Secondly, it has been revealed that particular microRNA species could be associated with specific etiology of stroke,^[51,54]^ while there has been no evidence for Hcy in this respect. Further, results from bioinformatics analysis show that in stroke some genes are noted to be targeted by microRNAs, which indicates a potential therapeutic target for the disease.^[51]^ However, several problems with microRNAs need to be resolved prior to their widespread application in clinical practice. Above all, compared to Hcy, detection of microRNAs is expensive and more complex thus necessitating exploration of an improved technique for analysis.^[50–52]^ The great number of species as well as their varying nature also brings about challenges in determining cut-off value for diagnosis.^[51]^ Additionally, the pathophysiological mechanism is not clear, and the therapeutic target is uncertain.^[50]^

Lipoprotein(a) has been demonstrated to be an independent risk factor for CVDs and niacin is therefore suggested as a possible treatment to reduce its level. However, there has been no success in robustly lowering its level in human study as yet.^[55,56]^

Adipokines including leptin, resistin, protein 4 (FABP4), and retinol binding protein 4(RBP4),^[57,58]^ have also been considered as risk factors associated with CVDs, stroke and hypertension.^[59–62]^ They are reported to be capable of inducing tissue factor, producing C-reactive protein,^[59,60]^ promoting insulin resistance^[63]^ and stimulating inflammatory reaction, oxidative stress, atherogenesis and thrombosis, resulting in endothelial dysfunction, arterial stiffness, and atherosclerotic plaques.

Lifestyle modifications as well as medications such as statins and hypoglycemics may be helpful in reducing adipokines levels.^[64]^ In this sense, adipokines are also promising tools for diagnosing CVDs that enable subsequent exploration of novel pharmacological interventions so long as a better appreciation of their function and molecular targets can be achieved.^[65]^

There are several limitations to our study. First, Hcy levels are affected by a variety of factors and we excluded subjects with a history of taking folic acid, vitamin B12, and other drugs that can influence Hcy levels. Second, as an observational study, we only measured the level of Hcy in 2000, so there is a lack of evaluation of the changes in the Hcy levels over the 17-year follow-up. Nevertheless, all participants were from the same geographical area and we were able to collect the information about stroke, CVDs, and hypertension. Third, the subjects were mostly males and elderly, and were from the Department of Geriatrics in Ruijin Hospital (Shanghai, China) and do not represent the whole population. Though, our analysis adjusted for confounders, majority of the included participants were of males and recent evidence suggests that the Hcy levels found be more in males compared to females. Therefore, the results should be for reference only and they need further confirmation. Finally, we did not further analyze the interaction between Hcy and hypertension.

## 5 Conclusions

Mildly elevated Hcy levels (10–15 μmol/L) are significantly associated with an increased risk for stroke and CVD events in this retrospective cohort study, and with new-onset hypertension without hypertension at baseline. Furthermore, Hcy improves the predictive capability of traditional risk factors for stroke. The optimal cut-off value of Hcy for predicting stroke, CVD, and hypertension was 13.4, 12.1, and 9.1 μmol/L, respectively, which might serve as reference values for future studies.

## Author contributions

**Conceptualization:** Wei Wang, Jiumei Cao.

**Investigation:** Yuanyuan Feng, Kai Kang, Qiqi Xue, Yafen Chen.

**Methodology:** Yuanyuan Feng, Kai Kang, Qiqi Xue, Yafen Chen.

**Writing – original draft:** Yuanyuan Feng, Jiumei Cao.

**Writing – review & editing:** Wei Wang, Jiumei Cao.
